# Initial Derivation of Positive Dementia-Related Observational Measures: A Descriptive Ethnography

**DOI:** 10.1093/geroni/igaa057.531

**Published:** 2020-12-16

**Authors:** Maya Staehler, Clark Benson, Jordan Madden, Laura Block, Andrea Gilmore-Bykovskyi

**Affiliations:** 1 University of Wisconsin-Madison School of Nursing, Madison, Wisconsin, United States; 2 Mayo Clinic, Rochester, Minnesota, United States; 3 University of Wisconsin-Madison, Madison, Wisconsin, United States

## Abstract

Person-centered caregiving approaches emphasize efforts to protect and maintain the personhood of people living with dementia (PLWD). The influence on person-centered caregiving approaches on PLWD have predominantly focused on deficit-oriented outcomes, such as absence or reduction of behavioral symptoms. While important to quality of life, the absence of measurable “positive” responses to person-centered caregiving approaches limit opportunities to specify sensitive and meaningful outcome measures that more holistically represent PLWD’s care experiences as more than the absence of a negative outcome. To address these gaps, we conducted a secondary analysis of video-observations of PLWD (N=9) surrounding mealtime cares using a descriptive ethnographic approach. Our objectives were to descriptively summarize specific responsive behaviors demonstrated by PLWD surrounding person-centered caregiving interactions, specifying observable features of these responses and consider their utility in future video-observational research. Findings indicate PLWD contribute both verbal and non-verbal communication surrounding person-centered approaches which can be characterized as conversational (starting conversation, answering or asking questions), expressing preferences (indicating needs and preferences, agreeing or disagreeing, complying with or refusing caregiver requests, permission granting), emotional responses (mirroring caregivers’ emotions, demonstrating emotion e.g. smiling), and reflexive (mirroring of caregiver’s actions), with overlap between categories. Findings suggest that PLWD not only contribute and respond in meaningful ways to person-centered interactions, but also initiate a significant number of these interactions. This study contributes to a growing body of research and advocacy that examines the personhood and abilities of PLWD and establishes the utility of observational data in studying PLWD contributions.

